# Method for Tunnel Displacements Calculation Based on Mobile Tunnel Monitoring System

**DOI:** 10.3390/s21134407

**Published:** 2021-06-27

**Authors:** Zeyu Yue, Haili Sun, Ruofei Zhong, Liming Du

**Affiliations:** Beijing Advanced Innovation Center for Imaging Theory and Technology, Key Laboratory of 3D Information Acquisition and Application, Ministry of Education, College of Resource Environment and Tourism, Academy for Multidisciplinary Studies, Capital Normal University, Beijing 100048, China; 2190902203@cnu.edu.cn (Z.Y.); zrf@cnu.edu.cn (R.Z.); limingado@163.com (L.D.)

**Keywords:** ring seam recognition, shield tunnel, radial displacement, circumferential displacement

## Abstract

Efficient, high-precision, and automatic measurement of tunnel structural changes is the key to ensuring the safe operation of subways. Conventional manual, static, and discrete measurements cannot meet the requirements of rapid and full-section detection in subway construction and operation. Mobile laser scanning technology is the primary method for tunnel detection. Herein, we propose a method to calculate shield tunnel displacements of a full cross-section tunnel. The point cloud data, obtained via a mobile tunnel deformation detection system, were fitted, projected, and interpolated to generate an orthophoto image. Combined with the cumulative characteristics of the tunnel gray gradient, the longitudinal ring seam of the tunnel was identified, while the Canny algorithm and Hough line detection algorithm identified the transverse seam. The symmetrical vertical foot method and cross-section superposition analysis were used to calculate the circumferential and radial displacements, respectively. The proposed displacement calculation method achieves automatic recognition of a ring seam, reduces human–computer interaction, and is fast, intelligent, and accurate. Furthermore, the description of the tunnel deformation location and deformation amount is more quantitative and specific. These results confirm the significance of shield tunnel displacement monitoring based on mobile monitoring systems in tunnel disease monitoring.

## 1. Introduction

In 2020, a total of 44 cities in China will put into operation 7715.31 km of urban rail transit lines. New lines and sections with a length of 1349.7 km have been put into operation in 26 cities, which presents a new record. With the rapid development of urban subways and high-speed railways, regular tunnel disease monitoring is essential to ensure tunnel safety.

The development of surveying and mapping technology is constantly modifying the operation methods of conventional measurement, such as the rapid development of three-dimensional (3D) laser scanning technology and vehicle mobile measurement systems, which play increasingly important roles in many fields with advantages such as high precision, speed mass, and non-contact measurement [1]. Mobile 3D laser scanning technology has been used in street view scanning [2], urban road extraction [3], road storage, building modeling, infrastructure management, and mobility evaluation [4,5,6], and has become an important and popular technology for modern surveying and mapping. Owing to characteristics such as high efficiency, good quality, and little influence from the surrounding environment, it is feasible to introduce mobile measurements into tunnels.

At present, the primary need for tunnel deformation monitoring is to obtain the vertical and horizontal displacements, cross-sectional and convergence deformations, cracks, and tunnel leakages to determine whether the tunnel has deformations, and subsequently, the deformation size and period. There are several reasons for tunnel displacement, including segment selection, shield machine propulsion speed, shield posture, and bolt hole diameter [7,8].

The mainstream tunnel deformation monitoring methods include total station and caliper measurement, with the measurement accuracy of the total station being a maximum of 1 mm. Based on the 3D coordinate non-contact measurement technology of free and automatic total stations, the corresponding mathematical adjustment model is established, and the average error of each 3D coordinate measurement point may reach a maximum of 0.8 mm [9,10,11]. However, owing to the large investment of surveyors and the complex environment within the tunnel, instrument operation is difficult and inefficient, making it difficult to achieve full section detection [12]. Targets for station-type 3D laser scanning need to be set, and an image mosaic is needed between adjacent stations. The scanning time for a single station is about 10 min, the data redundancy is large, and the working efficiency and post-processing is relatively low [13,14,15,16,17]. To overcome these disadvantages, mobile measurement equipment can be employed. Such equipment can quickly obtain high-quality 3D point clouds of the track and surrounding environment, mileage of the tunnel segment, accurate rail profile, and more information. It has flexible production operation coupled with high speed and image resolution, reliable data results, and comprehensive information [18]. The measurement speed of the mobile laser measurement system used in this study can reach a maximum of 5.4 km/h, which is 5–10 times higher than that of traditional measurement methods.

The current representative tunnel mobile measurement equipment is the GRP 5000 tunnel scanning measurement system developed by Amberg and Leica, known as SiTrack: One Mobile track scanning system [19,20]. Mobile tunnel inspection vehicles are also becoming popular in China, for example, the railway mobile measurement system (RMMS-01) developed by Wuhan University and the tunnel mobile measurement software and hardware integration system (TMMS) developed by Tongji University [21]. The TMMS for tunnel movement measurement developed by Tongji University and Wuhan University can fit the tunnel cross-section; however, it only supports the convergence diameter measurement in the clearance convergence measurement and cannot calculate the displacement. GRP 5000, SiTrack: One, and RMMS-01 are equipped with GNSS (Global Navigation Satellite System) positioning and inertial navigation systems, which can provide absolute coordinate measurement, but the hardware cost is high. The above four kinds of equipment are the mainstream mobile measurement systems in the market. However, some construction companies have customized the mobile measurement monitoring vehicles according to their own needs and develop control software only for their own use, for example, the tunnel mobile measurement system developed by the Shanghai Geotechnical Investigation and Design Engineering Consulting (Group) Co., Ltd. (Shanghai, China).

Tunnel deformation has always been a major problem affecting tunnel safety. Many scholars have studied tunnel deformation, such as the influence of ground building pile foundation on underground tunnel [22], installing support in tunnel soil layer to reduce tunnel deformation [23], or using convergence-confinement method to analyze tunnel deformation [24]. Some have also established mathematical models for the surrounding soil to analyze the settlement of the tunnel and the surface [25,26]. At this stage, many scholars have studied tunnel convergence measurements; however, our focus is on extracting the section center by tunnel section fitting and disease detection based on tunnel orthophoto images [27,28,29,30]. For example, the calculation of the tunnel convergence diameter based on point cloud data and the calculation of the shield tunnel convergence diameter by denoising, slicing, and ellipse fitting of the point cloud data. The calculation accuracy is approximately 2 mm [31,32]. Several studies have also explored the calculation of tunnel clearance, chord length, guide height, and pull-out value of the catenary based on mobile laser data and achieved a calculation accuracy of 3 mm [33]. An ultrasonic distance measuring instrument was installed in the same section of the shield tunnel to calculate the chord length of the tunnel, leading to a calculation accuracy of 2 mm [34]. According to the surrounding geological environment, some studies have established tunnel and surface building deformation prediction models, whose prediction accuracy can reach 75% [35,36]. The generation algorithm and quality evaluation of tunnel orthophoto images based on mobile laser systems have also been studied. This method uses orthophoto images for color classification and visualization of the deformation. Although this method intuitively reflects tunnel deformation, it cannot quantitatively reflect the deformation, which has certain limitations [37]. In engineering practice, manual inspection and caliper measurement are often used to check the displacement, hence the efficiency is low. At present, research on the calculation method and accuracy of shield tunnel displacements is still in the nascent stages. In this study, a mobile laser scanning system was used for data acquisition and an orthophoto image was used to achieve the automatic recognition of the circumferential and radial seam and the circumferential and radial displacements were calculated. Due to the limitation of hardware, the calculation method introduced in this paper is only applicable to the calculation of railway tunnels constructed by the shield method.

Next, we introduce the software and hardware of the mobile monitoring system. In Section 3, the generation method of orthophotos and the recognition method of circumferential gap are introduced as well as the method of calculation of radial and circumferential displacements. In Section 4, the precision of the calculation results is evaluated. In Section 5 and Section 6, future research prospects and suggestions, and the final research results of this paper are discussed.

## 2. Mobile Tunnel Monitoring System (MTDS)

**Figure 1:**
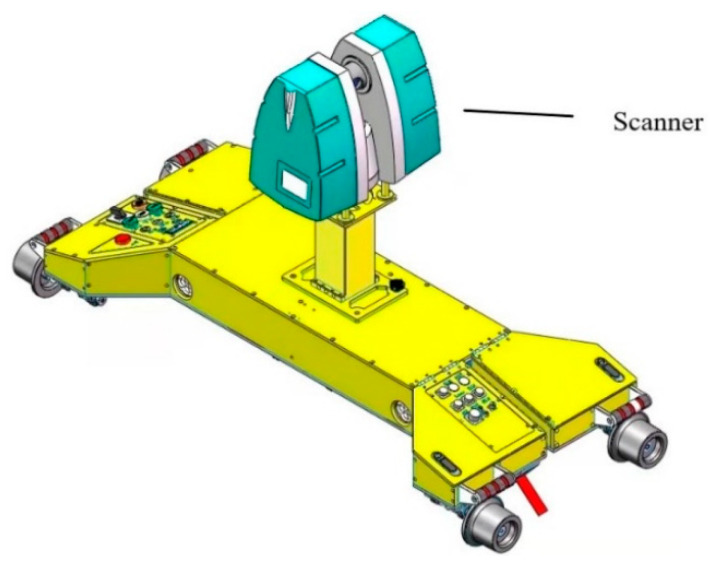
Schematic diagram of the mobile tunnel inspection vehicle.

The system mainly includes a scanner, mobile monitoring car body, and acquisition and processing software. The scanner employs three brands of scanners, namely, Faro, Leica, and Z + F, and supports the auxiliary mileage encoder. The equipment was driven by a motor having five gears with adjustable speed. The mobile tunnel monitoring system relies primarily on the scanners and mobile measuring vehicles for scanning. Further, a tunnel laser scanning acquisition and processing software was developed with a net native interface for the Z + F, Faro, and Leica scanners. By controlling the start and stop of the scanners and coordinating through the mobile monitoring equipment, laser scanning data can be quickly collected in the tunnel. This paper introduces some efficient algorithms, such as robust estimation least-squares, robust noise elimination, real-time signal processing algorithms, and surface computational geometry algorithms. Furthermore, we used a central processing unit (CPU) multithread parallel computing algorithm for data solution and point cloud rendering to ensure the overall efficiency and high precision of the software and achieve the integration of the tunnel point cloud data acquisition and post-processing. The system is illustrated in Figure 1.

Commonly used scanners in mobile monitoring systems are the Faro focus 3D 120, the Leica scanning P30/P40, and the Z + F PROFILER 9012. Their angle measurement accuracy are 19″, 8″, and 0.02°, respectively. Their ranging accuracy and scanning speed are ±2, ±1.2, and ±1 mm, and 97,600, 1,000,000, and 10,160,000 pixel/s, respectively.

## 3. Methods

**Figure 2:**
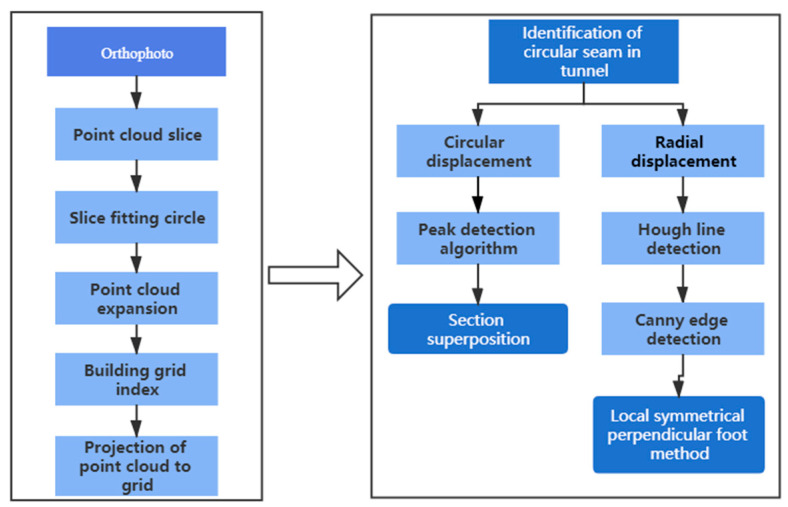
Flow chart of the displacement calculation method.

In this study we introduce an orthophoto generation algorithm, using orthophoto through peak detection algorithm, Canny edge detection, and Hough line detection algorithm to identify the capping block and longitudinal ring seam. Thereafter a cross-section superposition method is used to calculate the circular displacement using the local symmetrical perpendicular foot method to calculate the radial displacement. The calculation flowchart is shown in Figure 2.

### 3.1. Orthophoto Generation

#### 3.1.1. Point Cloud Fitting Circle

After denoising, the point cloud is sliced according to the mileage, with the forward direction of the measuring equipment as *y*. Thereafter, each slice can be approximated as a circle and regarded as a plane composed of *X* and *Z* coordinates. Denoising was followed by fitting the point cloud of each slice via the least-squares method, and the fitting results provided the center coordinates and the radius of the circle. The point cloud was projected according to the fitting results.

The definition equation of the circle is used to establish the model

(1) dist(P,A)+dist(P,B)=DIST

where *P* is a point on the circle and *A* and *B* are the foci. Three points on the circle were randomly selected to build the circle model, the distance between each point and the two foci as well as the difference between each point and the *DIST* was calculated. This point is in line with the model when the difference is less than a certain threshold. The model with the largest number of points is the best-circle model. According to the points that meet the conditions, the general equation of the circle is as follows,

(2) $$x_{i}^{2}+y_{i}^{2}+Ax+By+C=0,$$

Coefficient fitting was carried out according to the obtained coincidence points. For the circle fitting of the least-squares method, the optimal objective function of the error squared is:

(3) $$E=\overset{n}{\underset{i=0}{\sum }}{\left(x_{i}^{2}+y_{i}^{2}+{\mathrm{Ax}}_{i}+{\mathrm{By}}_{i}+C\right)}^{2},$$

**Figure 3:**
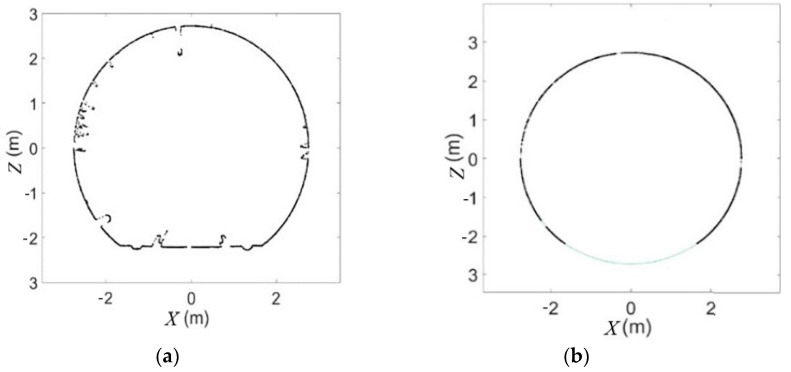
Before section fitting (**a**) and after section fitting (**b**).

According to the principle of the least-squares method, the parameters *A*, *B*, and *C* should be minimized. Finally, after the fitting the values best-fit center coordinates $\left(X_{0},Y_{0}\right)$ and radius *R* were obtained. The results are illustrated in Figure 3.

(4) $$X_{0}=-\frac{A}{2},Y_{0}=-\frac{B}{2},r=\frac{1}{2}\sqrt{A^{2}+B^{2}-4C},$$

#### 3.1.2. Point Cloud Projection According to the Tunnel Arc Length

After fitting the circle, each tunnel slice corresponded to a set of fitting circle parameters. Considering that the cloud coordinate of a point in the slice is (X,Z), the center coordinate of the circle is ($X_{R},Z_{R}$), and the radius of the fitting circle is *R*. In the next part of this paper, the tunnel slice is called tunnel section. The angle θ formed by the section points and the center coordinate of the circle uses the arctangent function as follows:

(5) $$\theta =\arctan\left(\frac{Y-Y_{R}}{X-X_{R}}\right)$$

Then according to the arc length formula of the circle:

(6) l=θR

**Figure 4:**
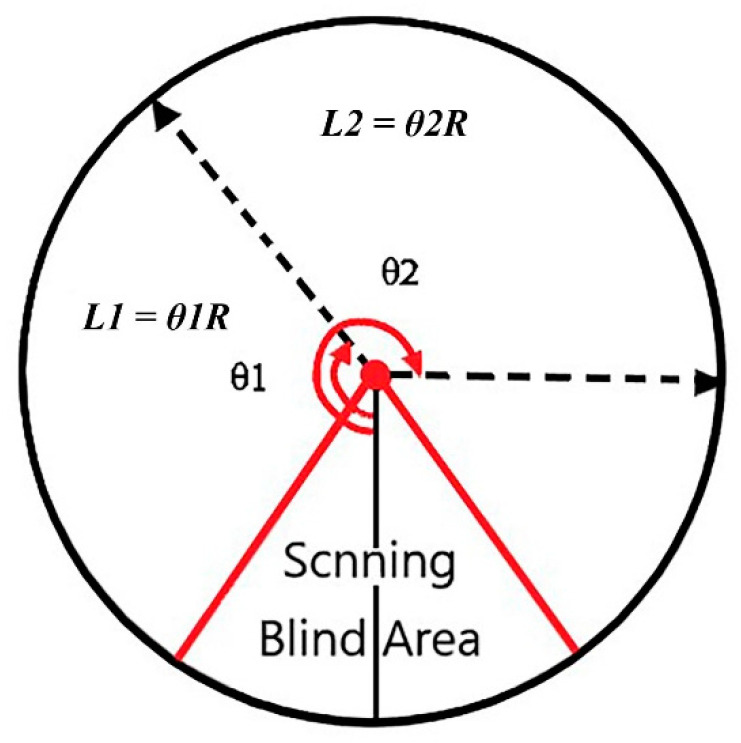
Schematic diagram of slice point cloud projection.

Therefore, each point in the slice cloud calculates the arc length *l* one by one, and each slice corresponds to the mileage m, thus the 3D coordinates are projected into plane coordinates (*m*, *l*). A point cloud expansion diagram is shown in Figure 4.

#### 3.1.3. Building Grid Index

In the last part, we have projected the coordinates of the point cloud to the plane. By traversing these points, we can find the maximum value $X_{max}$ and minimum value $X_{min}$ of the abscissa and the maximum value $Y_{max}$ and minimum value $Y_{min}$ of the ordinate. The plane grid is established according to the $X_{max}$, $X_{min}$ and $Y_{max}$, and $Y_{min}$. The grid width is set to 0.01 m. Thus, the values of the grid horizontal total number *a*, grid vertical total number *b*, and grid normalization parameter *n* (normalization of point cloud intensity) are as follows:

(7) $$a=\frac{X_{max}-X_{min}}{0.01}b=\frac{Y_{max}-Y_{min}}{0.01}n=\frac{1}{I_{max}-I_{min}}$$

The coordinates of the point cloud projected by the arc length in the upper section are recorded as $\left(X_{i},Y_{i}\right)$. At the same time, $\frac{X_{i}-X_{min}}{0.01}$ and $\frac{\left(Y_{i}-Y_{min}\right)}{0.01}$ are the *x*- and *y*-direction index value of the points, respectively. Through the establishment of the grid, every point in the point cloud is placed into the grid, and each point matches a corresponding grid index value.

#### 3.1.4. Grid to Image

An image matrix was established. The data type in the matrix was 64-bit single channel data, and the number of rows and columns were consistent with the grid index. The purpose of this step was to assign gray value to the image matrix. There are three scenarios in the projection.

1. There is only one point in the grid index
  If there is a point in the grid index corresponding to the row and column number of the matrix, and the intensity value of the point is *I*, then the gray value of the point $g=255\times \frac{I}{n}$ is stored in the row and column numbers corresponding to the matrix.
2. There are two or more points in the grid
  If there are two or more points in the grid, let the grid center coordinate be $\left(X_{0},Y_{0}\right)$ and the point coordinate be $\left(X_{i},Y_{i}\right)$. The Euclidean distance between the grid center and point was calculated as follows:
  (8) $$d=\sqrt{{\left(X_{0}-X_{i}\right)}^{2}+{\left(Y_{0}-Y_{i}\right)}^{2}}$$
  If there is a point such that $d<10^{-6}$, then the gray value $g=255\times \frac{I}{n}$ corresponding to that point is stored in the row and column numbers corresponding to the image matrix.
3. No point in grid corresponding to matrix
  If there are no points in the grid corresponding to the row and column numbers of the matrix, making $d<10^{-6}$, then the inverse distance weighted average interpolation (IDW) method was used. The reason of using IDW algorithm is to assign gray value to the grid (that is, image pixels) to prevent the existence of black spots in the image. The points in the point cloud data are regarded as discrete points in the plane, assuming that there are N discrete points $\left(X_{1},Y_{1}\right)$, $\left(X_{2},Y_{2}\right)$, …, $\left(X_{n},Y_{n}\right)$. The grid center is regarded as the predicted interpolation point Z(X,Y). The Euclidean distance formula was used as the distance function formula:
  (9) $$h_{i}=\sqrt{{\left(X-X_{i}\right)}^{2}+{\left(Y-Y_{i}\right)}^{2}}$$
  where (X,Y) is the interpolation point coordinate, and $\left(X_{i},Y_{i}\right)$ is the coordinate of each discrete point. When the power parameter is determined, the weight function is used to calculate the weight of each discrete point.
  (10) $$w_{i}=\frac{h_{i}^{-p}}{\sum_{i=1}^{n}h_{i}^{-p}}$$
  where *p* is the power parameter, and *n* is the number of discrete points; in this study, *p* = 3. The function for calculating the interpolation point is as follows:
  (11) $$Z\left(X,Y\right)=\overset{n}{\underset{i=1}{\sum }}w_{i}\times Z\left(X_{i},Y_{i}\right)$$
  where Z(X,Y) is the intensity value of the interpolation point, and the gray value *g* of the point is stored in the row and column number corresponding to the matrix. The calculation method for *g* is as follows:
  (12) $$g=255\times \frac{Z\left(X,Y\right)}{n}$$

**Figure 5:**
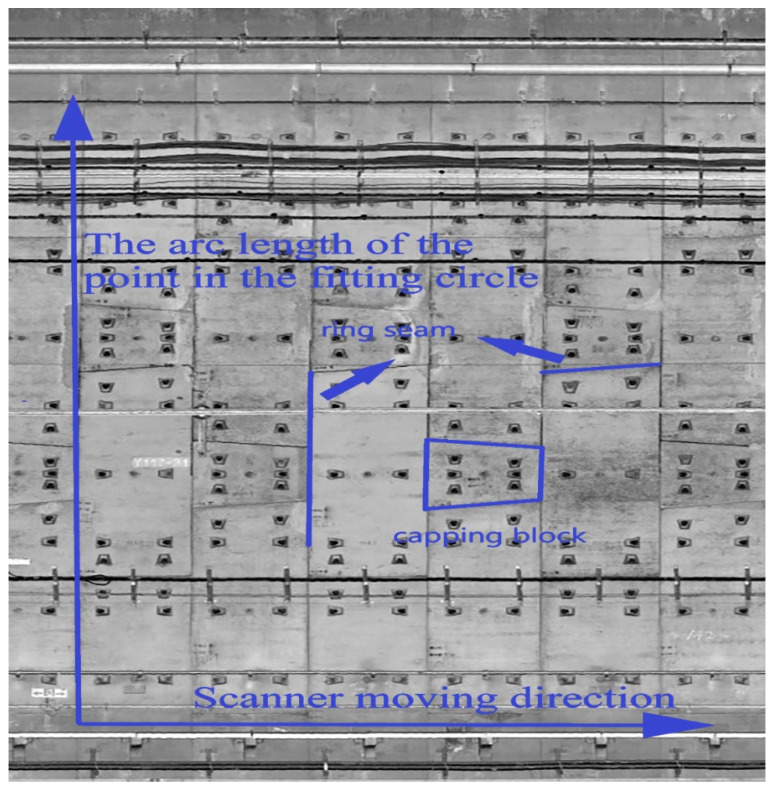
Orthophoto generation effect.

The gray orthophoto image in the tunnel can be obtained by outputting the image matrix. The orthophoto effect of the tunnel image is shown in Figure 5.

### 3.2. Longitudinal Ring Seam Identification

#### 3.2.1. Longitudinal Ring Seam Detection

In this study, the peak detection algorithm was used to detect the longitudinal ring seam, and the gray value of the orthophoto image was searched longitudinally. The tunnel seam is dark in the image (the gray value is small), and the seam between the rings is perpendicular to the *X*-axis. Using this feature, the automatic recognition of the longitudinal seam can be realized. First, the gray value difference between columns *i* + 1 and *i* − 1 column is calculated row by row according to the image matrix.

(13) $$\Delta G_{i}=G_{i+1}-G_{i-1},$$

**Figure 6:**
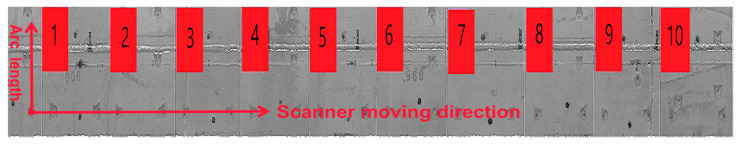
Recognition results of longitudinal ring seams.

After comparing *i* + 1 and *i* − 1 columns, the occurrence times of $\Delta G_{i}<0$ were counted as $t_{i}$, and the *t* value of each column of the matrix was recorded and treated as an array. Many peaks will appear in this array. According to the principle that the gray value at the ring seam is small and the longitudinal circumferential seam is perpendicular to the *X* axis, the column corresponding to the peak value is the position of the longitudinal ring seam. To avoid the influence of cracks and other ancillary tunnel facilities, the data of 0.75 m on either side of the first determined ring joint were not processed, thus eliminating identification errors. The above steps were repeated, and after several iterations, all the ring joints were identified; however, there is a possibility of gross errors. Therefore, we used the root mean square error (RMSE) to eliminate gross errors. Let $x_{1}$, $x_{2}$, …, $x_{n}$ be the column index of the calculated longitudinal ring seams, and *md* be the arithmetic average of the interval column numbers between the longitudinal ring seams.

(14) $$md=\frac{x_{1}+x_{2}+\ldots +x_{n}}{n}$$

(15) $$Z=\sqrt{\frac{\sum_{i=1}^{n}{\left(x_{i}-md\right)}^{2}}{n}},$$

where *Z* is the root mean square of $x_{1}$, $x_{2}$, …, $x_{n}$. If $\left|x_{i}-md\right|>2Z$ is satisfied, then if *I* is not the corresponding column number of the longitudinal ring seam, it will be eliminated. After the circular seam is identified, the image pixel position is inversely indexed to the point cloud section data such that the position of the ring seam in the point cloud can be determined. The results of the longitudinal ring seam identification are shown in Figure 6. Through annular seam detection, the orthophoto image can be stored in different rings, which is convenient for later transverse seam detection and tunnel displacement calculation.

#### 3.2.2. Transverse Seam Inspection of Capping Block

1. Canny image binarization

**Figure 7:**
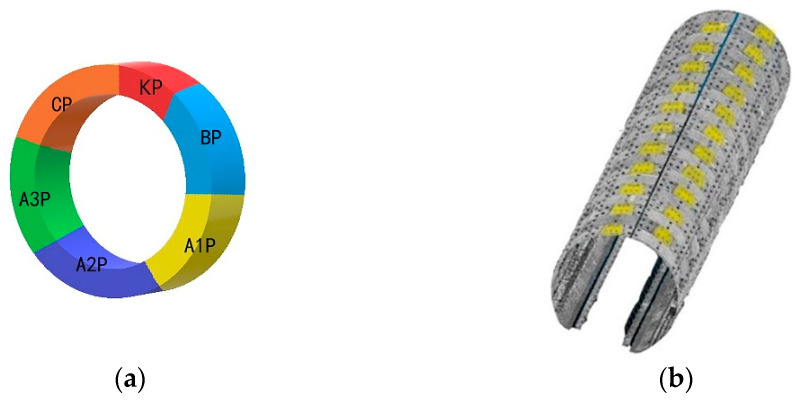
(**a**) Schematic segmentation diagram. (**b**) Capping block (KP) position in point cloud.

Each shield tunnel ring consists of six segments, one capping block (KP), two adjacent blocks (CP, BP), and three standard blocks (A1p, A2p, A3p). According to the design data of the tunnel, the segment and installation angles of the capping block can be determined. The block diagram of the tunnel segment is shown in Figure 7a, and the position of the tunnel capping block is shown in Figure 7b. According to the corresponding angle of the capping block in the tunnel, the point cloud data can be preprocessed, and the orthophoto image of the capping block can be generated to prevent the interference of other tunnel ancillary facilities.

In this study, the Canny operator was used for image binarization. At first, the Canny operator used the Gaussian filtering method to smoothen the pixels in the image neighborhood, and thereafter the pixels in different positions in the neighborhood were assigned different weights.

**Figure 8:**
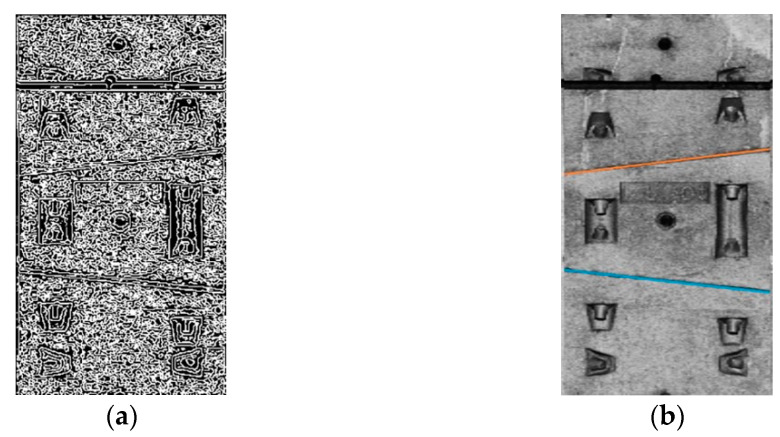
(**a**) Canny edge detection result. (**b**) Hough line detection result.

While smoothing the image, most of the overall gray distribution characteristics of the image are retained. The 3D Gaussian distribution function is expressed as follows:

(16) $$G\left(x,y\right)=\frac{1}{2{\pi \sigma }^{2}}e^{-\frac{x^{2}+y^{2}}{2\sigma^{2}}},$$

where σ represents the standard deviation, and the squares of $S_{x}$ and $S_{y}$ represent the distance between other pixels and the center pixel in the neighborhood, respectively. Subsequently, the gradient operator (Sobel) was used to calculate the gradient amplitude and direction:

(17) $$G\left(x,y\right)=\sqrt{S_{x}^{2}+S_{y}^{2}}$$

(18) $$R\left(x,y\right)=\arctan\left(\frac{S_{x}}{S_{y}}\right)$$

where $S_{x}$ and $S_{y}$ are the convolutions of the gray level and grayscale in the *X* and *Y* direction, of the original image, respectively, G(x,y) is the gradient amplitude, and R(x,y) is the gradient direction. The edge intensity and direction at each point were estimated by calculating the gradient amplitude and direction. According to the gradient direction, the gradient amplitude was non-maximally suppressed, and finally, the double threshold method was used to detect and remove certain noise and false edges. The edge detection result of the Canny operator is shown in Figure 8a.

2. Hough line detection

Hough line detection transforms the line in the image space to a point in the parameter space and solves the detection problem through statistical characteristics. Specifically, if the pixels in an image form a straight line, then the curves corresponding to the pixel coordinate values (*x*, *y*) in the parameter space must intersect at a point, such that we only transform all the pixel coordinate values in the image into curves in the parameter space, and then detect the intersection of the curves in the parameter space to determine the straight line. The expression for the Hough parameter space is as follows:

(19) $$r=x_{i}\cos\theta +y_{i}\sin\theta ,$$

Theoretically, a point corresponds to innumerable lines in the parameter space; however, in practical applications, the number of lines (that is, a limited number of directions) must be limited for calculation. Therefore, a straight line can be drawn by discretizing *θ*, calculating *r* according to the coordinates (*x*, *y*) of the point, counting the occurrence times of (*R*, *θ*), and selecting the two (*R*, *θ*) values that were repeated the most. In this study, Hough line detection was used to identify the two transverse ring seams of the capping block. According to the segment design data, the ring seam position information of the other four segments can be inferred. The results of the Hough line detection are shown in Figure 8b.

### 3.3. Tunnel Displacement Calculation

#### 3.3.1. Tunnel Radial Displacement Calculation

The radial displacement of shield tunnel refers to the phenomenon that the inner walls of adjacent segments in the same ring are not on a curved surface after the tunnel segments are assembled. In this study, the local symmetrical vertical foot method is used to solve the tunnel radial displacement. According to the tunnel transverse joint detection results, the real point cloud coordinates of the capping block circumferential joints are obtained via reverse retrieval of the point cloud. The middle position of the longitudinal circumferential seam can better reflect the radial dislocation of the segment. Thus, the section in the middle of the transverse joint (the section in the middle of the tunnel segment) is selected to calculate the radial displacements. The joint positions of other segments are calculated from the segment design data. Five adjacent sections in the middle of the circumferential seam are selected for circle fitting using the least square method, and five center coordinates $\left(X_{0},Y_{0}\right)$ are obtained. Each section can be regarded as a circle in the plane Cartesian coordinate system. Taking the capping block joint as an example, we set the joint position point coordinate as *A*$\left(X_{A},Y_{A}\right)$, make a straight line from the center of the circle to the capping block joint position and perform the same operation for the five sections. Then, the functional expression of the line is as follows:

(20) $$\frac{x-X_{A}}{X_{0}-X_{A}}=\frac{y-Y_{0}}{Y_{A}-Y_{0}}$$

**Figure 9:**
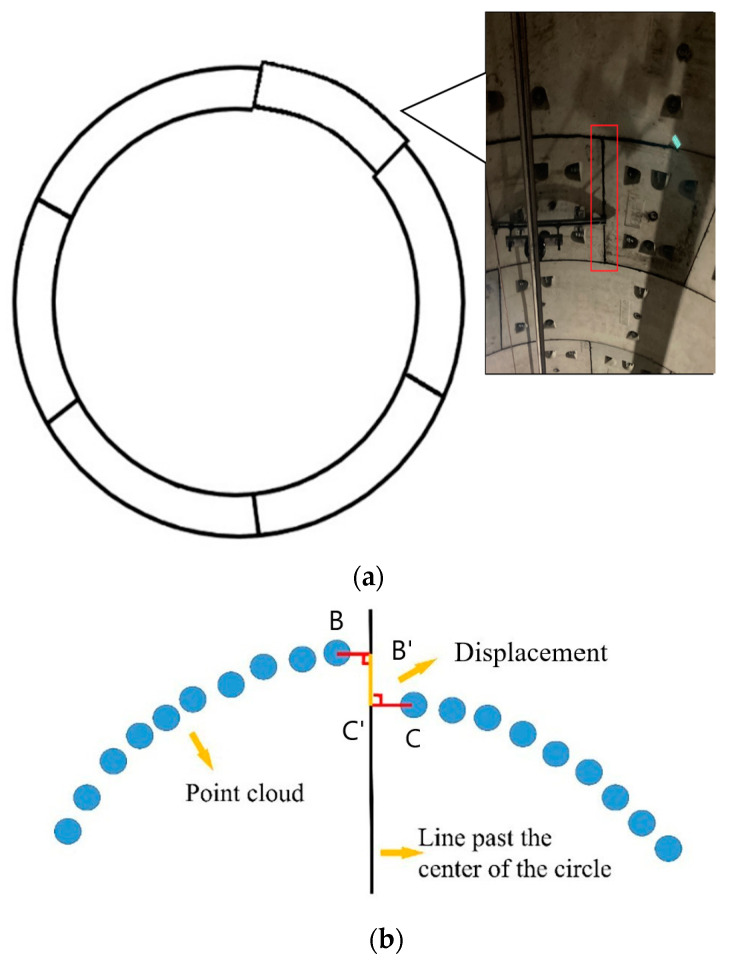
(**a**) Schematic radial displacement diagram and site photo of radial dislocation. (**b**) Local symmetric foot drop method.

Furthermore, we select the nearest point on the left side of point *A* as *B* and the point on the right side of point *A* as *C*. To prevent the influence of noise points, we do not require point *B* and point *C*. Therefore, we need to set a threshold. The distance difference between points *B* and *C* and joint position point *A*$\left(X_{A},Y_{A}\right)$ should be less than 10 cm. Points *B* and *C* lead the perpendicular foot to the straight line $\frac{x-X_{A}}{X_{0}-X_{A}}=\frac{y-Y_{0}}{Y_{A}-Y_{0}}$ (the straight line from the center of the circle to the position of the joint obtained in the previous step), and the perpendicular foot point is marked as $B^{\prime }$ and $C^{\prime }$, respectively. The distance between the two perpendicular feet is the displacement value between the two segments, as shown in Figure 9b. The same calculation method was used for the five adjacent sections and an average value was obtained. The same process is followed for the other transverse joints, for example, BP–A1P. The radial displacement diagram is shown in Figure 9a.

#### 3.3.2. Circumferential Displacement Calculation

**Figure 10:**
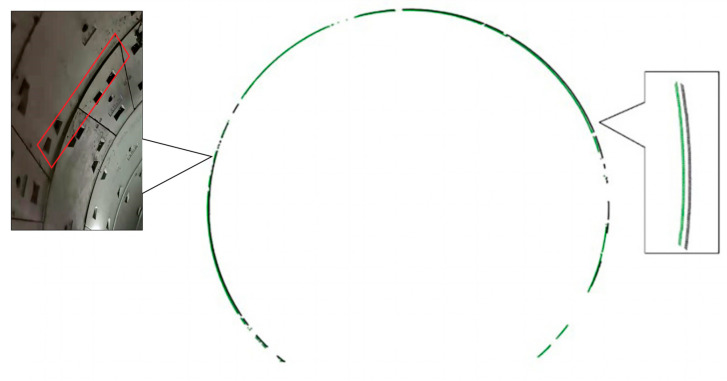
Sectional superposition results.

The circumferential displacement of the shield tunnel is the phenomenon of displacement between two adjacent rings in the forward direction of the tunnel after the tunnel segments are assembled. Section superposition method is used to calculate circumferential displacement. According to the longitudinal ring detection results, the position information (odometer value) of the circumferential seam is obtained. In the point cloud, the nearest 10 adjacent sections on the left and right sides of the longitudinal ring seam are retrieved. After eliminating noise points, the first section on the left side of the circumferential seam is selected for circle fitting to obtain the section center $\left(X_{0},Y_{0}\right)$ and radius *R*. Then, a section is found on the corresponding symmetrical position on the right side of the circumferential seam. The two sections are superposed according to the center of the fitting circle. The superposition result is shown in Figure 10. The distance from each point on the left and right section to the fitting center, respectively, is calculated. The distance is calculated as:

(21) $$d=\sqrt{{\left(X_{0}-X_{i}\right)}^{2}+{\left(Y_{0}-Y_{i}\right)}^{2}}$$

The distance from each point to the center of the left section is subtracted from the distance from the corresponding point of the right section to the center of the circle. If the difference between the distance *d* and the fitting radius *R* is more than 5 mm, and the arc length of the continuous displacement with a difference exceeding 5 mm is more than 0.5 m, the starting and ending angles of the displacement, the arc length of the displacement, and the arithmetic mean value of each displacement segment are counted. In the previous step, 10 sections were taken from the left and right sections, and a total of 10 groups of data were calculated in the same way. The average value of 10 sets of data is output as the result of circumferential displacement. The schematic cross sectional superposition diagram is shown in Figure 10.

## 4. Method Validation

### 4.1. Data Sources

**Figure 11:**
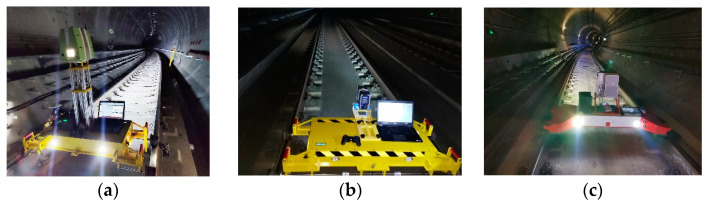
Operation photos of (**a**) Leica (**b**) Faro (**c**) Z + F.

Data from Tianjin and Nanchang Metro systems were used in this verification. The Tianjin metro tunnel is a circular shield tunnel with a ring width and inner diameter of 1.5 and 5.5 m, respectively. The tunnel was put into operation for operation safety monitoring. The Nanchang metro tunnel is also a circular shield tunnel with ring width and inner diameter of 1.5 and 5.4 m, respectively. The tunnel has been put into operation. The tunnel in the test section was built many years ago. Due to the displacement of the shield tunnel segments caused by geological changes, there are circumferential and radial displacements. The circumferential displacement is calculated using the section superposition method, and the radial dislocation is calculated using the local symmetric foot method. The mobile monitoring vehicle travels at a constant speed in the tunnel. Simultaneously, the acquisition software is used to control the start and stop of the scanner. The same vehicle speed and the same type of scanner were used in the same tunnel section for repeated scanning. Leica P16, Faro Focus 3D 120 and Z + F 9012 scanners (as shown in the Figure 11) were used for scanning. Leica P16, Faro, and Z + F 9012 operation photos are shown in Figure 11a–c, respectively.

### 4.2. Circumferential Displacement Round-Trip Precision Verification

In this study, Nanchang metro data were used for circumferential displacement precision verification. Circumferential displacement is the displacement of two adjacent rings in the mileage direction and the calculation result represents the average value of the displacement of two adjacent rings. Circumferential displacement is calculated by section superposition method. The length of the tunnel used for the experiment was 50 m. The mobile tunnel measurement system with Leica P16 was used to carry out the round-trip measurement at a speed of 0.5 m/s, and the round-trip comparison of 35 groups of circumferential displacement was carried out. The maximum absolute deviation of the round-trip measurement was 2.9 mm, and the arithmetic mean deviation was 1.4 mm. The results are shown in Table 1.

### 4.3. Radial Displacement Round-Trip Precision Verification

**Figure 12:**
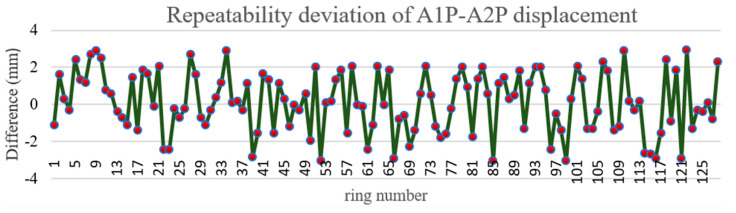
Repeatability deviation of A1P–A2P displacement.

**Figure 13:**
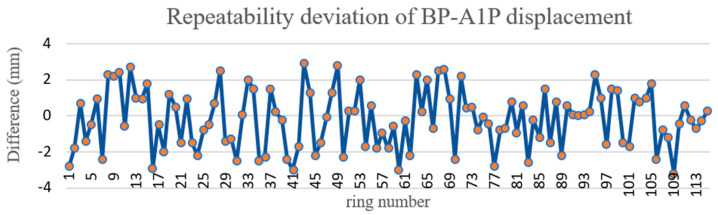
Repeatability deviation of BP–A1P displacement.

**Figure 14:**
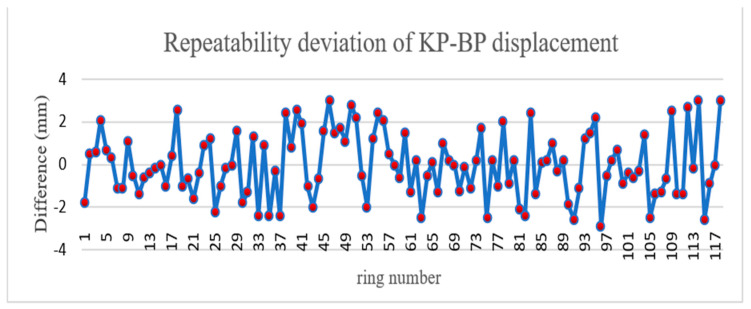
Repeatability deviation of KP–BP displacement.

The Tianjin test data were used to verify the repeatability precision of radial displacement. Radial displacement is the phenomenon that the inner walls of adjacent segments in the same ring are not on the same arc surface and stagger with each other. The shield tunnel wall consists of six segments, one top block (KP), two adjacent blocks (CP, BP) and three standard blocks (A1p, A2p, A3p). The radial displacement is calculated by the vertical foot method. The test length was approximately 200 m. Because there is cable shielding in the Tianjin tunnel, only KPBP, BP–A1P, and A1P–A2P were used to verify the repeatability precision of radial displacement. The A1P–A2P, BP–A1P, and KP–BP displacements are shown in Figure 12, Figure 13 and Figure 14, respectively. According to the comparison of round-trip data, the KP–BP round-trip maximum deviation was 3.0 mm with an average deviation of 1.2 mm, the BP–A1P round-trip maximum deviation was 3.0 mm with an average deviation of 1.4 mm, while the A1P–A2P round-trip maximum deviation was 3.0 mm with an average deviation of 1.3 mm. The round-trip measurement precision was within 3.0 mm.

### 4.4. Radial Displacement Comparative Precision Verification

**Figure 15:**
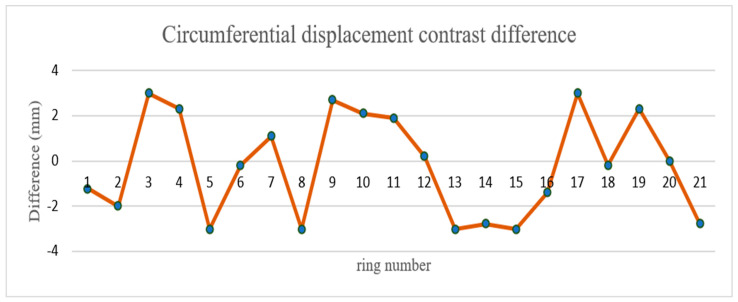
Circumferential displacement contrast difference.

**Figure 16:**
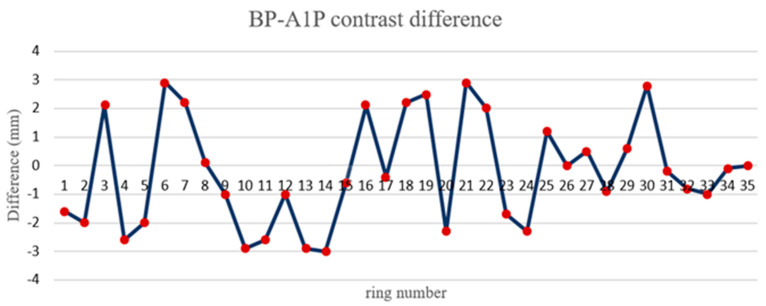
BP–A1P contrast difference.

**Figure 17:**
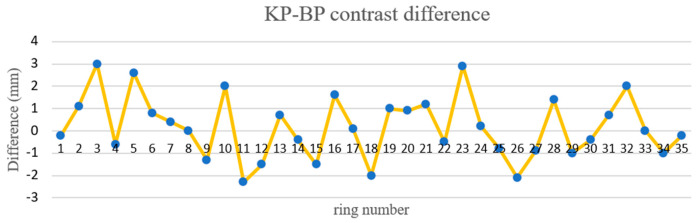
KP–BP contrast difference.

In this study, Focus 3D 120 and Z + F 9012 scanners were used to scan the 50 m shield tunnel of Tianjin Metro. The purpose of this experiment was to use different brands of scanners to scan in the same tunnel and compare the difference. Using this data, 21 groups of radial dislocation and 35 groups of circumferential displacement were calculated. The circumferential displacement was verified by KP–BP and BP–A1P. The difference of circumferential displacement is shown in Figure 15, and that of radial displacement is shown in Figure 16 and Figure 17. According to the comparison of data from different brands of scanners, the maximum circumferential displacement difference was 3.0 mm with an average difference of 1.96 mm, the KP–BP maximum difference was 3.0 mm with an average difference of 1.12 mm, and the BP–A1P maximum difference was 3.0 mm with an average difference of 1.6 mm. The contrast difference precision was within 3.0 mm.

### 4.5. Radial Displacement Absolute Accuracy Verification

Tianjin data were used to verify the absolute accuracy of the radial displacement algorithm. Because the BP–A1P ring seam was close to the ground and easy to measure, the BP–A1P ring seam was used to verify the absolute accuracy. In this study, 10 groups of BP–A1P radial displacement were measured using vernier calipers and compared with the radial displacement calculated by the local symmetrical vertical foot method. The comparison results are shown in Table 2. The maximum radial displacement difference was 3.0 mm with an average difference of 1.98 mm.

This study used three methods to verify the precision of the algorithm, which are round-trip repeatability precision verification, comparison verification of different brands of scanners in the same tunnel section, and absolute accuracy verification of vernier calipers measurement value compared with the measurement value of this method. These results showed that the calculation accuracy of the circumferential and radial displacements achieved good results. Through the test comparison, the average value of repeatability difference of circumferential displacement was 1.4 mm, the average value of repeatability difference of three groups of radial displacement was 1.3 mm, the difference of circumferential displacement contrast using different scanners was 1.96 mm, and the average difference of radial displacement contrast between the two groups was 1.36 mm. The absolute accuracy of radial displacement verified by vernier calipers was within 3 mm. The maximum difference of all validation was less than 3 mm, which meets the Metro deformation monitoring precision requirements.

## 5. Discussion

In this paper, we proposed a displacement calculation method based on mobile laser scanning, which includes circumferential and radial displacement calculation. Tunnel displacement monitoring plays an important role in current tunnel clearance convergence measurement. At present, the traditional manual inspection method, vernier caliper measurements, total station measurements, and model predictions are mostly used for tunnel displacement monitoring. However, the data acquisition speed of these technologies is low and the information obtained is not comprehensive. Furthermore, they do not provide a quantitative displacement calculation; therefore, data acquisition of full section displacements cannot be achieved. In this paper, the mobile laser scanning method was used to achieve rapid tunnel data acquisition and generate high-definition tunnel orthophotos. The tunnel ring seam recognition algorithm greatly reduces the in-office data processing workload. In the calculation of circumferential displacement, the section superposition method was used to comprehensively evaluate the displacement of two adjacent rings and calculate and output the average displacement. When calculating the radial displacement, the local symmetrical vertical foot method was used to evaluate the displacement between different segments in the same ring. Due to the shielding of tunnel cables, pipelines, and other ancillary facilities, some radial displacement could not be accurately calculated. Because the mobile monitoring vehicle can only run on the track, there is no in-depth study on its applicability. This paper mainly studies the structural deformation calculation of shield tunnel, without in-depth study on the applicability of the platform. However, the calculation method mentioned in this paper is suitable for shield tunnels with various purposes, which is only limited by hardware equipment (such as road tunnel). In future, the platform can be improved to drive on the ground, and the applicability of the algorithm can be explored. Despite its many advantages, the method proposed in this paper also has some limitations, such as the need to obtain tunnel segment design data, image quality dependence, the tunnel point cloud quality and the tunnel design data have a greater dependence, some capping block contours are not sufficiently clear, and the ring seam recognition effect is not ideal. To overcome these limitations and further improve the recognition effect, a deep learning neural network could be used in future work for the capping block and circumferential seam identification.

## 6. Conclusions

The calculation method proposed in this paper enriches the existing content of tunnel convergence measurement. Combining the orthophoto generation algorithm, peak detection algorithm, image Canny edge detection, and Hough line detection algorithm, the automatic recognition of tunnel ring seams and tunnel capping blocks was achieved. The section superposition and local symmetrical vertical foot methods were used to calculate the circumferential displacement and radial displacement, respectively. The experimental results showed that the repeatability precision of the circumferential and radial displacements was within 3 mm and the results of the difference comparison of circumferential and radial displacement using different scanners and the absolute accuracy verification using vernier calipers were less than 3 mm. The mining method, shield method construction monitoring and operation line track, track bed, concrete structure, engineering structure in construction, tunnel vault sinking, and structure convergence deformation, with an allowable error of ±3 mm, met the accuracy requirements. At present, the calculation of tunnel displacement was always the key and difficult point in tunnel deformation detection. The section superposition method and the local symmetrical vertical foot method proposed in this paper can accurately and comprehensively evaluate the tunnel shape variables, which provides a guarantee for the construction safety and operational safety. The calculation accuracy of the method can achieve good results through comparison. Although the total station method has higher accuracy in tunnel deformation monitoring, this method has lower efficiency and cannot meet the requirement of data acquisition speed. Some studies have explored the convergence diameter calculation method based on the mobile laser scanning method achieving good results; however, the specific segment displacement location cannot be determined. Other studies have established deformation prediction models through the geological environment around the tunnel; however, their prediction accuracy is not ideal. Based on the mobile laser scanning system, the point cloud data in the tunnel can be obtained quickly and conveniently, in contrast with the traditional contact measurement method. The orthophoto image was generated from the point cloud data, and an image processing algorithm was used to automatically identify the position of the circumferential seam and the position of the capping block. Finally, the point cloud data were retrieved in a reverse manner for the calculation of tunnel displacement, which quickly provided large and accurate tunnel displacement data for tunnel monitoring work, greatly reducing the field workload and human–computer interaction, improving the operation efficiency. The current algorithm was heavily dependent on the image quality, the quality of the tunnel point cloud, and the tunnel design data. Furthermore, the recognition effect is unsuitable for certain capping blocks and circumferential seams whose contours are not clear enough. To further improve the recognition effect, we propose the use of deep learning neural networks to recognize the capping blocks and circumferential seams.

## Figures and Tables

**Table 1:** Circumferential displacement round-trip precision comparison.

Number	Forward Displacement (mm)	Backward Displacement (mm)	Absolute Deviation (mm)
1	7.7	10.1	2.4
2	8.7	9.4	0.7
3	9.7	12.1	2.4
4	7.1	9.9	2.8
5	12.5	10.7	1.8
6	9.1	9.3	0.2
7	10.8	14.0	3.2
8	9.9	8.6	1.3
9	7.2	10.1	2.9
10	7.5	9.1	1.6
11	10.8	10.5	0.3
12	15.7	18.0	2.3
13	10.1	9.6	0.5
14	7.8	7.8	0.0
15	11.2	12.0	0.8
16	8.6	9.3	0.7
17	7.7	9.1	1.4
18	8.7	8.0	0.7
19	11.2	13.8	2.6
20	7.7	9.5	1.8
21	12.1	11.3	0.8
22	13.2	11.4	1.8
23	12.1	12.4	0.3
24	8.0	7.8	0.2
25	9.8	7.4	2.4
Absolute Mean Deviation (mm)			1.4

**Table 2:** Radial displacement absolute accuracy comparison.

Number	Measured Value of Vernier Calipers (mm)	Radial Displacement Measured by Proposed Method (mm)	Absolute Deviation (mm)
1	3.5	4.5	1.2
2	5.3	3.0	2.3
3	2.1	5.1	3.0
4	7.4	4.9	2.5
5	1.5	3.7	2.2
6	3.8	6.0	2.2
7	4.9	2.2	2.7
8	1.9	1.6	0.3
9	3.6	6.1	2.5
10	3.2	4.1	0.9
Absolute Mean Deviation (mm)			1.98

## Data Availability

Not applicable.
